# Studies on tracheorelaxant and anti-inflammatory activities of rhizomes of *Polygonatum verticillatum*

**DOI:** 10.1186/1472-6882-13-197

**Published:** 2013-07-29

**Authors:** Haroon Khan, Muhammad Saeed, Malik Hassan Mehmood, Najeeb-ur Rehman, Naveed Muhammad, Ikram-ul Haq, Nadeem Ashraf, Kamal Eldin H El-Tahir, Anwarul-Hassan Gilani

**Affiliations:** 1 Department of Pharmacy, University of Peshawar, Peshawar 25120, Pakistan; 2 Natural Product Research Division, Department of Biological and Biomedical Sciences, The Aga Khan University Medical College, Karachi 74800, Pakistan; 3 National Institute of Health, Islamabad 45500, Pakistan; 4 Gandhara College of Pharmacy, Gandhara University, Peshawar, Pakistan; 5 Molecular Brain Research Group, Robarts Research Institute, Department of Physiology & Pharmacology, Faculty of Medicine, University of Western Ontario, 100 Perth Drive, London, ON N6A 5K8, Canada; 6 Department of Pharmacology, College of Pharmacy, King Saud University, Riyadh, Saudi Arabia; 7 Department of Basic Medical Sciences, Faculty of Pharmacy, Gomal University, D.I. Khan-29050, Khyber-Pakhtoonkha, Pakistan; 8 Department of Pharmacy, Hazara University, Havelian Campus, Abbottabad, Pakistan

**Keywords:** Polygonatum verticillatum, Bronchodilator, Ca^2+^ antagonist, Anti-inflammatory, 2- Hydroxybenzoic acid, β-sitosterol

## Abstract

**Background:**

The present study describes the tracheorelaxant and anti-inflammatory effects of *Polygonatum verticillatum* which may support its medicinal use in hyperactive airway complaints and inflammatory disorders.

**Methods:**

The tracheorelaxant activity of crude extract of the rhizomes of *P. verticillatum* (PR) was assessed in isolated guinea-pig tracheal tissues immersed in tissue organ bath filled with Tyrode’s solution and a continuous supply of carbogen gas (95% O_2_ and 5% CO_2_). The contractile and relaxant responses of the tissue were measured using isometric transducers coupled with Power-Lab data acquisition system. The anti-inflammatory effect was evaluated in carrageenan-induced rat paw edema model, while the lipoxygenase inhibitory activity was performed in the *in-vitro* assay*.* Various chromatographic and spectroscopic techniques were used for the isolation and characterization of pure molecules.

**Results:**

In isolated guinea-pig tracheal preparations, PR caused complete inhibition of the high K^+^ (80 mM) and carbachol-induced contractions however, it was more potent against K^+^ than CCh, similar to verapamil. Pretreatment of the tissue with PR, displaced the Ca^2+^ concentration-response curves to the right, similar to that induced by verapamil, indicating the presence of Ca^2+^ channel blocking like activity. When tested on carrageenan-induced rat paw edema*,* PR demonstrated a marked reduction in edema with 65.22% protection at 200 mg/kg, similar to aspirin. In the *in-vitro* assay, PR showed lipoxygenase inhibitory activity (IC_50_: 102 ± 0.19 μg/mL), similar to baicalein. Bioactivity-guided fractionation led to the isolation of 2-hydroxybenzoic acid and β-sitosterol.

**Conclusions:**

These results indicate that the plant possesses tracheorelaxant, mediated possibly through a Ca^2+^ channel blockade mechanism, and anti-inflammatory activities, which may explain the medicinal use of this plant in airway disorders and inflammation.

## Background

*Polygonatum verticillatum* [L.] All. (Nooreallam) belonging to family *Liliaceae* or *Convallariaceae* possesses around 57 species and is commonly found in East Asia, China and Japan [1,2]. In different traditional systems of medicine, *Polygonatum* is popular for its use in pulmonary disorders like asthma and inflammation [3,4], in addition to its multiple other health benefits such as, antituberculant, antidiabetic, antihypertensive, diuretic, analgesic and antipyretic activities [4,5]. *P. verticillatum* has also been studied for its analgesic [6,7], antimalarial and antioxidant [8], metal accumulant [9], insecticidal [10], antibacterial [11] and antipyretic [12] activities.

A variety of phytochemical constituents have been isolated from different species of the genus *Polygonatum*; primarily: saponins, alkaloids, glycosides, flavonoids and phytohormones. These groups of compounds show different types of activities. Long chain esters from this plant exhibit potent tyrosinase inhibition [13], alkaloid, homoisolflavanone, triterpenoid and steroidal saponin show profound antimicrobial and anticancer properties [14-17] and emodin from *Polygonatum* has shown ameliorating effects on the memory consolidation [18]. Several compounds have also been isolated from the rhizomes of *P. verticillatum* including lectins [19], 5-hydroxymethyl-2-furaldehyde and diosgenin [8]. There is no study to the best of our knowledge reporting its usefulness in hyperactive airways disorders or inflammation. This study describes the tracheorelaxant and anti-inflammatory activities of *P. verticillatum* to provide a scientific background to its medicinal use in hyperactive airways complaints like asthma or inflammatory conditions. The *in-vivo* and *in-vitro* experimental studies have been designed, followed by bioactivity-guided isolation of its secondary metabolites.

## Method

### Plant material

*P. verticillatum* (whole plant) was collected from the District Swat, Khyber Pukhtonkhawa, Pakistan, in July-Aug 2007. The botanical characterization of the plant material was executed by the Taxonomy Department of PCSIR Laboratories Peshawar and a specimen with catalogue No: 9970 (PES) was deposited in the herbarium of PCSIR Laboratories Peshawar.

### Plant extraction

Air-shade dried rhizomes of the *P. verticillatum* (8 kg) were grounded to a fine powder. The powdered material was soaked in aqueous-methanol (30:70) for three days while shaking occasionally [20] and filtered through a muslin cloth and Whatman filter paper (Maidstone, UK) simultaneously. This procedure was repeated three times and all the pooled filtrates were evaporated on a rotary evaporator (model RE-111, Buchi, Flawil, Switzerland) under reduced pressure (−760 mm Hg) to obtain a dark greenish semi-solid material, yielding 27.50 wt/wt%.

### Experimental animals

A total of thirty Wistar rats (190–260 g) and five adult local guinea-pigs (1.2 − 1.6 kg) of either sex were kept under standard laboratory conditions at 25 ± 2°C and a relative humidity of 40-70%. The light cycle was maintained as 12 h dark: 12 h light. They were fed with a laboratory diet *ad libitum* and allowed free access to drinking water. All the experiments were performed in compliance with the rulings of the Institute of Laboratory Animal Resources, Commission on Life Sciences, National Research Council [21] and approved by the local Ethical Committee of the Karachi University.

### Drugs and reagents

Carrageenan, carbachol (CCh), verapamil hydrochloride, soybean lipoxygenase, linoleic acid sodium salt and baicalein were purchased from the Sigma Chemicals Co., St. Louis, MO, USA and aspirin was obtained from the Reckitt & Colman, Pakistan. Chemicals used for making Tyrode’s solution were: potassium chloride (Sigma Chemicals Co., St. Louis, MO, USA), calcium chloride, glucose, magnesium chloride, sodium bicarbonate, sodium dihydrogen phosphate (Merck, Darmstadt, Germany) and sodium chloride from BDH Laboratory supplies, Poole, England. All chemicals used were of highest grade available, and were solubilized in distilled water/saline while carrageenan was used as suspension with acacia.

### Isolated guinea-pig tracheal tissue

The trachea was obtained from guinea-pigs and preserved in physiological solution (Kreb’s solution). Rings containing a couple of cartilages were formed from a tracheal tube approximately 2–3 mm wide. Rings were cut into strips by a longitudinal cut on the ventral side reverse to the smooth muscle [22]. The strips were suspended in a tissue bath (20 mL) containing Kreb’s solution (pH 7.4), maintained at 37°C and aerated with a mixture of 95% oxygen and 5% carbon dioxide (carbogen). Tracheal strips were maintained at 1 gram constant tension during the course of experiment. The tissues were granted 1 h to equilibrate prior to the introduction of test material. Before determining the inhibitory activity of the plant extract, the isolated tracheal tissues were stabilized with high K^+^ (80 mM) and CCh (1 μM) until constant responses of each agonist were achieved (usually 3–4 concentrations). The sustained contractions were obtained using CCh and K^+^ separately and the inhibitory effect was assessed using the cumulative addition of the test material. Isometric responses were recorded on a Grass model 7 Polygraph (Grass instrument company, Quincy, MA, USA). The inhibition of the high K^+^-induced contractions indicates Ca^2+^ antagonist activity [23], which was confirmed by constructing the Ca^2+^ concentration-response curves in the absence and presence of increasing concentrations of the plant material.

### Carrageenan-induced edema

The carrageenan-induced rat hind paw edema test was conducted as described previously [24]. The test animals were divided in to five groups (*n* = 6). Group Ι (the control group) received normal saline (10 mL/kg). The rats of group ΙΙ, ΙΙΙ and ΙV received the test extract (50, 100 or 200 mg/kg i.p.), while, group V (positive control) received aspirin (100 mg/kg i.p.). Following 30 min of the treatments, acute inflammation was induced by sub-plantar injection of 0.1 mL of 1% suspension of carrageenan with 2% gum acacia in normal saline, in the right hind paw of the rats. The paw volume was estimated with the help of plethysmometer (Ugo Basile, Italy) at 1^st^, 2^nd^, 3^rd^, 4^th^ and 5^th^ h after the carrageenan injection. Statistics was applied on the raw data for the calculation of reduction in rat paw volume (mL) for each group against saline, followed by the calculation of percent reduction in the rat paw using the following formula:

$$\%\;\mathrm{inhibition}=1-\left(\mathrm{dt}/\mathrm{dc}\right)\times 100$$

where “dt” is the difference in paw volume in the treated group and “dc” the difference in paw volume in the control group.

### Soybean lipoxygenase inhibitory assay

The lipoxygenase (LOX) inhibition assay was conducted by using different dilutions of the PR by following previously described method [25]. Soybean lipoxygenase and linoleic acid were used. Equal volume (10 mL) of the sample (PR) and standard drug along with 20 mL of solvent lipoxygenase solution simultaneously were mixed followed by incubation for 5 min at 25°C. The biochemical reaction was initiated by the addition of linoleic acid solution (10 μL) as substrate and the absorption change with the formation of (9Z,11E)-13S)-13-hydroperoxyoctadeca-9, 11-dienoate was followed for 10 min at 234 nm. The test sample and the control were dissolved in 50% ethanol. All the reactions were performed in triplicate. Baicalein was used as a positive control. The median effective concentrations (IC_50_ values) were calculated using the EZ-Fit Enzyme Kinetics program (Perrella Scientific Inc., Amherst, USA).

### Isolation of pure molecules

A sample of 80 g of the aqueous-methanol extract of *P. verticillatum* rhizomes (PR) was subjected to column chromatography over silica gel (column grade) for the isolation of pure chemical compounds. The sample was loaded in a glass column over silica gel for adsorption that acts as stationary phase. The column was initiated with 100% *n*-Hexane as eluent (mobile phase). Polarity of mobile phase was enhanced gradually with regular monitoring of the isolate status over TLC, it resulted in 12 sub-fractions (P1-P12). Similar with methanol/chloroform gradient, 9 subfractions (M1-M9) were obtained. When sub-fraction P9 was purified through column chromatography, 2-Hydroxybenzoic acid (A) was isolated in the colorless crystals form (1A). Subfraction P11 was re-chromatographed over silica gel. While eluting with chloroform: hexane; (8:2), β-Sitosterol (B) was isolated as colorless amorphous powder (1B).

### Characterization of 2-Hydroxybenzoic acid (A)

Colorless crystals, M.P. 159-160°C, HREIMS: *m/z* 138 (Calcd. for C_7_H_6_O_3_; 138.12), ^1^H NMR (CHCl_3,_ 300 MHz): δ 6.89 broad d (*J* = 8.4 Hz), 7.42 dt (*J* = 1.8, 8.4 Hz), 6.85 t (*J* = 7.8 Hz), 7.83 dd (*J* = 1.8, 8.4), ^13^C NMR (CDCl_3_, 75 MHz): δ 113.9 (C-1), 163.2 (C-2), 118.1 (C-3), 136.6 (C-4), 120.0 (C-5), 131.5 (C-6), 173.5 (C-1′).

### Characterization of β-sitosterol (B)

Colourless amorphous powder, M.P. 135-136°C, HREIMS: *m/z* 414.3845 (calculated for C_29_H_50_O as 414.3855). ^1^H NMR (CHCl_3,_ 300 MHz): δ 3.65 (m), 5.35 (m), 0.67 (s), 0.99 (s), 0.91 d (*J* = 6.3 Hz), 0.83 d (*J* = 6.8 Hz), 0.79 d (*J* = 6.8 Hz), 0.82 t (*J* = 7.0 Hz). ^13^C NMR (CDCl_3_, 75 MHz): δ 37.1 (C-1), 31.6 (C-2), 72.1 (C-3), 42.4 (C-4), 139.5 (C-5), 120.0 (C-6), 32.5 (C-7), 35.3 (C-8), 49.8 (C-9), 36.2 (C-10), 22.1 (C-11), 40.3 (C-12), 43.1 (C-13), 56.9 (C-14), 24.7 (C-15), 27.9 (C-16), 54.7 (C-17), 12.1 (C-18), 18.7 (C-19), 40.1 (C-20), 21.5 (C-21), 33.8 (C-22), 28.8 (C-23), 50.5 (C-24), 26.7 (C-25), 18.9 (C-26), 21.5 (C-27), 23.1 (C-28), 12.6 (C-29).

### Statistical analysis

Results obtained from the pharmacological experiments are expressed as mean values ± S*.*E.M. One-way ANOVA test was employed for comparison of the significant differences among the groups followed by Dunnet’s multiple comparison post-test. A probability of *P <* 0*.*05 was considered as significant. Concentration–response curves were analyzed by nonlinear regression using GraphPad program (GraphPAD, San Diego, CA, USA).

## Results

### Effect of PR on guinea-pig tracheal tissue

**Figure 1:**
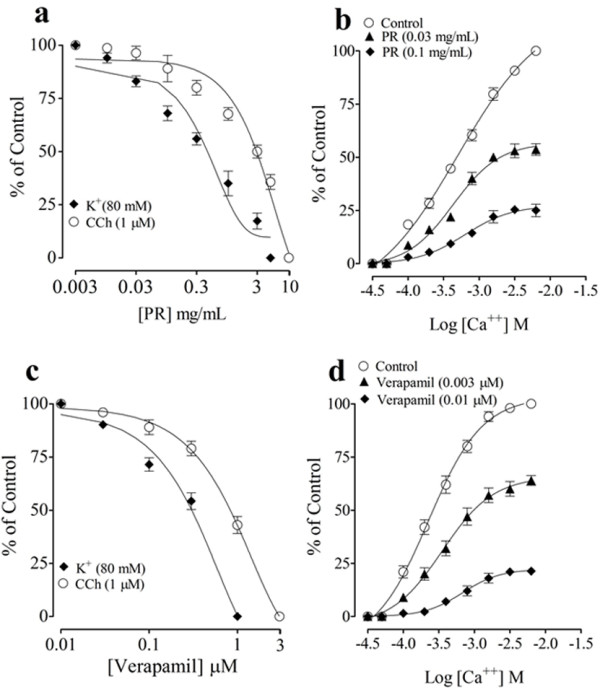
**Dose-dependent inhibitory effect of the aqueous-methanol extract of *P. verticillatum *Rhizomes, PR (a) and verapamil (c) on carbachol (CCh) and K**^**+ **^**(80 mM)-induced contractions, and the dose–response curves of Ca**^**2+ **^**created in the absence and presence of increasing concentrations of PR (b) and verapamil (d) in isolated guinea-pig jejunum preparation.** Data stand for mean ± SEM of 3–5 different experimental findings.

When PR was studied for its inhibitory effect against high K^+^ (80 mM) and CCh (1 μM) induced contractions in guinea-pig tracheal preparations, it caused inhibitory effects at the dose range of 0.01-10 mg/mL in a dose-dependent manner with greater potency against K^+^ (Figure 1a), similar to that of verapamil, which exhibited relaxation at the dose range of 0.03-3 μM (Figure 1c).

To confirm the Ca^2+^ antagonist-like effect, the concentration-response curves (CRCs) of Ca^2+^ were constructed in the absence and presence of different doses of the plant extract, in Ca^2+^-free and K^+^ rich medium. Pretreatment of PR at the doses of 0.03 and 0.1 mg/mL, displaced the CRCs of Ca^2+^ to the right with suppression of the maximum response (Figure 1b), similar to that caused by verapamil, which also displaced the Ca^2+^ CRCs to the right with suppression of the maximum response at tested doses of 0.003 and 0.01 μM (Figure 1d).

### Effects of PR on carrageenan-induced rat paw edema

PR demonstrated marked reduction (*P* < 0.01) in edema showing anti-inflammatory activity at the test doses of 50, 100 and 200 mg/kg, similar to that caused by aspirin (Table 1). The data show that the effect is dose and time-dependent with a peak effect obtained after 3 h of administration of PR (200 mg/kg), similar to the effect of aspirin.

**Table 1:** **Anti-inflammatory effect of the aqueous-methanol extract of *P. verticillatum *** **Rhizomes (PR) in carrageenan-induced hind paw edema in rats**

**Group**	**Dose mg/kg**	**Increase in paw volume (Mean ± SEM) in mL**				
		**1 h**	**2 h**	**3 h**	**4 h**	**5 h**
**Saline**	**10**	0.70 ± 0.031	0.69 ± 0.040	0.69 ± 0.049	0.70 ± 0.067	0.72 ± 0.053
**PR**	**50**	0.64 ± 0.045 (08.57%)	0.61 ± 0.054 (11.59%)	0.55 ± 0.049 (20.29%)	0.54 ± 0.053 (22.85%)	0.55 ± 0.049 (23.61%)
	**100**	0.56 ± 0.036 (20.00%)	0.50 ± 0.049* (27.53%)	0.37 ± 0.058** (46.37%)	0.39 ± 0.062** (44.29%)	0.47 ± 0.062* (34.72%)
	**200**	0.47 ± 0.040* (32.86%)	0.35 ± 0.067** (49.27%)	0.24 ± 0.045** (65.22%)	0.31 ± 0.053** (55.71%)	0.34 ± 0.062** (52.78%)
**Aspirin**	**100**	0.23 ± 0.040** (67.14%)	0.19 ± 0.017** (72.46%)	0.17 ± 0.031** (78.1%)	0.18 ± 0.022** (75.71%)	0.18 ± 0.036** (75.00%)

Experimental data are expressed as mean ± S.E.M. for group of at least six animals. One- way ANOVA was utilized as judgment test of significant differences among groups followed by Dunnett’s multiple comparison post-test. A probability of ^*^P < 0.05 or **P < 0.01 was considered significant from control. Protection (%) is shown in parenthesis.

### Effect of PR on lipoxygenase activity

When tested for inhibition of soybean lipoxygenase by the UV absorbance based enzyme assay, PR showed a significant activity against lipoxygenase with resultant IC_50_ value of 102 ± 0.19 μg/mL (mean ± SEM, n = 3) compared with that of the standard drug, baicalaim (22.6 ± 0.09 μg/mL, n = 3).

### Isolation of pure molecules

**Figure 2:**
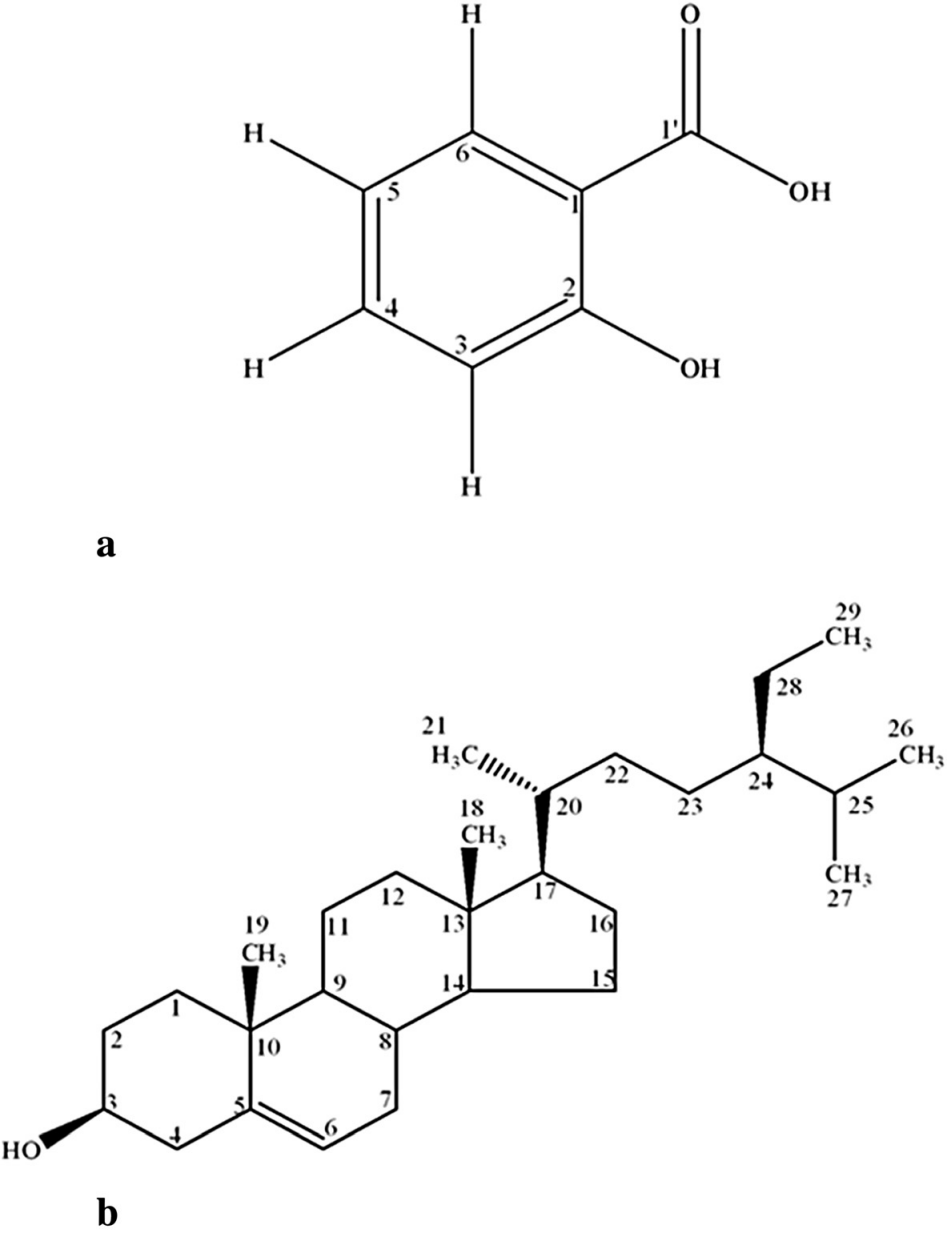
Structures of isolated compounds (a) 2-hydroxybenzoic acid, (b) β-sitosterol.

The structures of isolated molecules, 2-hydroxybenzoic acid (a) and β-sitosterol (b) were confirmed by mass and NMR spectral data available in literature [26,27] and shown in Figure 2.

## Discussion

In view of its medicinal use in hyperactive airway disordered like bronchial spasms and asthma [3], *P. verticillatum* has been studied for its myorelaxant activity using isolated guinea-pig tracheal tissue preparations. While on the basis of its folk reputation as an anti-inflammatory remedy [4], the plant has been tested for its anti-inflammatory potential using carrageenan-induced rat paw edema model.

When test for tracheorelaxant activity, *P. verticillatum* caused relaxation of high K^+^ and CCh-induced contractions in isolated guinea-pig tracheal tissues with greater potency against K^+^. The smooth muscle contraction of different body systems including the airways depends upon an increase in the cytoplasmic free Ca^2+^, which activates the cellular contractile machinery. The increase in intracellular Ca^2+^ is due to either influx via voltage-dependent Ca^2+^ channels (VDCs) or release from intracellular stores in the sarcoplasmic reticulum. At concentrations higher than 30 mM, K^+^ is known to cause smooth muscle contractions through the opening of VDCs, consequently allowing inward movement of extracellular Ca^2+^ originating a contractile effect; thus, a substance causing inhibition of high K^+^ -induced contraction is considered as an inhibitor of Ca^2+^ influx [23]. Similar to the pattern of inhibitory effect of verapamil, a standard Ca^2+^ antagonist [28], against K^+^ and CCh, a substance causing inhibition of high K^+^ at low concentrations than its effect on CCh, may indicate the presence of Ca^2+^ antagonist-like spasmolytic mechanism. To confirm the Ca^2+^ antagonist-like effect of *P. verticillatum*, the concentration-response curves (CRCs) of Ca^2+^ were constructed in the absence and presence of different doses of the plant extract, where it displaced the CRCs of Ca^2+^ to the right with suppression of the maximum response similar to the effect produced by verapamil, thus attesting the presence Ca^2+^ antagonist-like constituents in the plant extract. The Ca^2+^ antagonists are known to possess therapeutic potential in the management of hyperactive airways disorders [29,30]. Thus, the Ca^2+^ antagonist effect observed in this study may explain the medicinal use of the plant in airways disorders, though additional mechanism cannot be ruled out.

The carrageenan-induced paw edema test is a well-established animal model of inflammation used to detect the anti-inflammatory activity of test materials [25]. It has been observed that the local edema is induced by the sub-plantar injection of carrageenan that increases progressively. Edema formation due to carrageenan injection in the rat paw is the biphasic event during 1–5 h; the initial phase (1 to 1.5 h) is predominately a non-phagocytic edema followed by a second phase with increased edema formation that persists up to 5 h [31,32]. Different mediators are known to be involved in different stages of carrageenan-induced edema. The initial phase (up to 1.5 h) is attributed to the release of histamine, 5-hydroxytryptamine, bradykinin, platelet activating factor and serotonin. Kinins (bradykinin and kallikrein) are involved in both stages, and get released from 1.5 to 2.5 h and at the last step inflammation is continued until 5 h due to the release of lipid derived eicosanoids (prostaglandins, leukotrienes, hydroperoxyeicosatetraenoic acid). A comprehensive phagocytic inflammation is observed at 3^rd^ h followed by carrageenan injection with large number of neutrophils and tissue edema [25,31]. Our results showed significant (*P* < 0.01) inhibition in carrageenan-induced paw edema model at all test doses of anti-inflammatory activity of *P. verticillatum* with maximum effect at 3^rd^ h. The observed anti-inflammatory activity provides an evidence to the folkloric use of *P. verticillatum* in inflammation.

Leukotrienes (LTs) are the downstream products of arachidonic acid that exert pivotal biological functions as well as pathogenic effects in a wide range of inflammatory processes. Polymorphonuclear leukocytes and monocytes/macrophages are the major cells capable of synthesizing LTs due to a high 5-Lipoxygenase activity and represent crucial components in chronic inflammatory diseases [33-35]. The role of lipoxygenase is also documented in carrageenan provoked edema [33]. Inhibition of lipoxygenase activity results in down regulation of the pro-inflammatory activity of leukocytes and platelets [36], which may cause a diminished or delayed outcome of the inflammatory reaction. From a mechanistic point of view, PR elicited marked inhibitory activity in soybean lipoxygenase assay.

Phytochemically, the isolation of 2-hydroxybenzoic acid and β-sitosterol strengthened our findings in a way that the anti-inflammatory and antioxidant potential of these compounds have been previously investigated [37-39]; the effect of 2-hydroxybenzoic acid is known to be mediated through lipoxygenase (LOX)/cyclooxygenase (COX) inhibition [40,41]. Keeping this in view, it can also be speculated that the mechanism underlying the anti-inflammatory activity of *P. verticillatum* is at least in part due to LOX/COX inhibition, however the additional mechanism cannot be ruled out which may be responsible for the effectiveness of this plant in hyperactive airways disorders.

Free radical generation plays a pivotal role in the pathophysiology of inflammatory and airways disorders [42,43]. *P. verticillatum* is known to possess strong anti-oxidant activity [8], which is likely to complement the anti-inflammatory potential of this plant. Similarly, β-sitosterol is known to possess the Ca^2+^ channel blocking activity [22], which is again consistent with our findings, showing Ca^2+^ antagonist activity of *P. verticillatum*.

There is paucity in traditional literature regarding exact dose of the plant recommended to be used in airways disorders or as an external anti-inflammatory agent; however, the observed effective doses were selected on the basis of our experience of studying the possible mode of actions of natural remedies for their effectiveness in airways disorders and inflammatory conditions [12,44-46].

## Conclusions

These data indicate that *P. verticillatum* possesses tracheorelaxant, mediated possibly through the Ca^2+^ channel blockade and anti-inflammatory activities, which may explain the medicinal use of this plant in airway disorders and inflammatory complaints. However, detailed studies are warranted to ascertain the clinical significance of this natural product.

## Competing interests

The authors declare that they have no competing interests.

## Authors’ contributions

MS, AHG and KEHET designed the project, supervised the study and draft the final version of manuscript. NR, IKH and NM helped in the draft, experimental work, data collection and evaluation, literature search and manuscript preparation. MHM helped in study design, analysis of data and preparation of manuscript draft. HK and NA carried out experimental work and prepared initial draft of the manuscript All authors read and approved the final manuscript for publication.

## Pre-publication history

The pre-publication history for this paper can be accessed here:

http://www.biomedcentral.com/1472-6882/13/197/prepub

## References

[B1] Tamura MN Biosystematic studies on the genus polygonatum (liliaceae) 111. Morphology of staminal filaments and karyology of elevan Eusasian species. Botanische Jahrbücher für Systematik 1993 115 1 26

[B2] Monika S Jakub S Kornelia P Czeslaw H Roman Z Comparison of three *Polygonatum* species from Poland based on DNA markers Ann Bot Fennici 2006 43 378 388

[B3] Jiang S Dictionary of Chinese herbal medicines 1977 Shanghai: Shanghai People’s Publishing Press 2041 2044

[B4] Duke JA Bogenschutz-Godwin MJ Du Celliar J Duke PAK Handbook of medicinal herbs 2002 Boca Raton FL: CRC Press 683

[B5] Singh AP Ashtavarga–rare medicinal plants Ethnobot Leaflets 2006 10 104 108

[B6] Khan H Saeed M Gilani AH Khan MA Dar A Khan I The antinociceptive activity of *Polygonatum verticillatum* rhizomes in pain models J Ethnopharmacol 2010 127 521 527 19853648 10.1016/j.jep.2009.10.003

[B7] Khan H Saeed M Gilani AH Khan MA Khan I Ashraf N Anti-nociceptive activity of aerial parts of *polygonatum verticillatum*: attenuation of both peripheral and central pain mediators Phytother Res 2011 25 1024 1030 21254271 10.1002/ptr.3369

[B8] Khan H Saeed M Khan MA Khan I Ahmad M Muhammad N Khan A Antimalarial and free radical scavenging activities of rhizomes of *Polygonatum verticillatum* supported by isolated metabolites Med Chem Res 2011 21 1278 1282

[B9] Saeed M Khan H Khan MA Khan F Khan SA Muhammad N Quantification of various metals accumulation and cytotoxic profile of aerial parts of *Polygonatum verticillatum* Pak J Bot 2010 42 3995 4002

[B10] Saeed M Khan H Khan MA Simjee SU Muhammad N Khan SA Phytotoxic, insecticidal and leishmanicidal activities of aerial parts of *Polygonatum verticillatum* Afri J Biotech 2010 9 1241 1244

[B11] Khan H Saeed M Muhammad N Ghaffar R Khan SA Hassan S Antimicrobial activities of rhizomes of *Polygonatum verticillatum*: attributed to its total flavonoidal and phenolic contents Pak J Pharm Sci 2012 25 463 467 22459478

[B12] Khan H Saeed M Gilani AH Ikram-ul H Ashraf N Najeeb-ur R Haleemi A Antipyretic and anticonvulsant activity of *Polygonatum verticillatum*: comparison of rhizomes and aerial parts Phytother Res 2013 27 468 471 22610947 10.1002/ptr.4721

[B13] Khan H Saeed M Khan MA Izhar-ul H Muhammad N Ghaffar R Isolation of long chain esters from the rhizome of *Polygonatum verticillatum* with potent tyrosinase inhibition Med Chem Res 2013 22 5 2088 2092

[B14] Wang Y Lu C Lai G Cao J Luo S A new indolizinone from *Polygonatum kingianum* Planta Med 2003 69 1066 1068 14735451 10.1055/s-2003-45160

[B15] Wang D Li D Zhu W Peng P A new C-methylated homoisoflavanone and triterpenoid from the rhizomes of *Polygonatum odoratum* Nat Prod Res 2009 23 580 589 19384735 10.1080/14786410802560633

[B16] Wang D Li D Zhu W Zhang J Peng P Steroidal saponins from the rhizomes of *Polygonatum odaratum* Nat Prod Res 2009 23 940 947 19521907 10.1080/14786410902750977

[B17] Rafi MM Vastano BC Identification of a structure specific Bcl-2 phosphorylating homoisoflavone molecule from Vietnamese coriander (*Polygonatum odoratum*) that induces apoptosis and G2/M cell cycle arrest in breast cancer cell lines Food Chem 2007 104 332 340

[B18] Lu MC Tsuen HM Rei WC Yuan CH Hsieh CC Lin YT Peng WH Ameliorating effect of emodin, a constitute of *Polygonatum multiflorum*, on cycloheximide-induced impairment of memory consolidation in rats J Ethnopharmacol 2007 112 552 556 17572029 10.1016/j.jep.2007.05.004

[B19] Antoniuk VO Purification and properties of lectins of Polygonatum multiflorum [L.] All. and *Polygonatum verticillatum* [L.] All Ukr Biokhim Zh 1993 65 41 8 8351740

[B20] Williamson EM Okpako DT Evans FJ Selection, preparation and pharmacological evaluation of plant material Pharmacological methods in phytotherapy research 1996 Volume 1 Chichester: John Wiley and Sons 15 23

[B21] National Research Council Guide for the care and Use of laboratory animals 1996 Washington: National Academy Press 1 7

[B22] Gilani A Khan A Raoof M Ghayur M Siddiqui B Vohra W Begum S Gastrointestinal, selective airways and urinary bladder relaxant effects of *Hyoscyamus niger* are mediated through dual blockade of muscarinic receptors and Ca^2+^ channels Fund Clin Pharmacol 2008 22 87 99 10.1111/j.1472-8206.2007.00561.x 18251725

[B23] Gilani AH Mandukhail SU Iqbal J Yasinzai M Aziz N Khan A Rehman NU Antispasmodic and vasodilator activities of *Morinda citrifolia* root extract are mediated through blockade of voltage dependent calcium channels BMC Complement Altern Med 2010 10 2 20070879 10.1186/1472-6882-10-2 PMC2829485

[B24] Yim JH Lee OH Choi UK Kim YC Antinociceptive and anti-Inflammatory effects of ethanolic extracts of *Glycine max* (L.) Merr and *Rhynchosia nulubilis s*eeds Int J Mol Sci 2009 10 4742 4753 10.3390/ijms10114742 20087462 PMC2808008

[B25] Khan S Mehmood MH Ali ANA Ahmed FS Dar A Gilani AH Studies on anti-inflammatory and analgesic activities of betel nut in rodents J Ethnopharmacol 2011 135 654 661 21501676 10.1016/j.jep.2011.03.064

[B26] Jadrijevi M Taka M FT-IR and NMR spectroscopic studies of salicylic acid derivatives. I. Gentisamide–a metabolite of salicylamide Acta Pharm 2004 54 163 176 15610614

[B27] Moghaddam F Farimani M Salahvarzi S Amin G Chemical constituents of dichloromethane extract of cultivated constituents of dichloromethane extract of cultivated *Satureja khuzistanica* Evid Based Compl Altern Med 2007 4 95 98 10.1093/ecam/nel065 PMC1810368 17342246

[B28] Fleckenstein A Specific pharmacology of Ca^2+^ in myocardium, cardiac pacemakers and vascular smooth muscle Rev Pharmacol Toxicol 1977 17 149 166 10.1146/annurev.pa.17.040177.001053 326161

[B29] Mathewson HS Anti-asthmatic properties of calcium antagonists Respir Care 1985 30 779 781

[B30] Iwata S Ito S Iwaki M Kondo M Sashio T Takeda N Sokabe M Hasegawa Y Kume H Regulation of endothelin-1-induced interleukin-6 production by Ca^2+^ influx in human airway smooth muscle cells Eur J Pharmacol 2009 605 15 22 19171135 10.1016/j.ejphar.2008.12.045

[B31] Khan H Khan MA Muhammad N Ashraf N Gul F Tariq SA Antiinflammatory and antioxidant activity of Joshanda partially mediated through inhibition of lipoxygenase Phytopharmacol 2012 3 19 28

[B32] Jean YP Michael HP Steven BA Prostaglandin E2 synthesis and secretion: the role of PGE2 syntheses Clin Immunol 2006 119 229 240 16540375 10.1016/j.clim.2006.01.016

[B33] Thorsten JM Tausch L Hoernig M Coste O Schmidt R Angioni C Metzner J Groesch S Pergola C Steinhilber D Werz O Geisslinger G Celecoxib inhibits 5-lipoxygenase Biochem Pharmacol 2008 76 862 872 18692027 10.1016/j.bcp.2008.07.009

[B34] Geroushi A Auzi AA Elhwuegi AS Elzawam F Elsherif A Nahar L Sarker SD Antiinflammatory sesquiterpenes from the root oil of *Ferula hermonis* Phytother Res 2011 25 774 777 21520471 10.1002/ptr.3324

[B35] Viji V Helen A Inhibition of lipoxygenases and cyclooxygenase-2 enzymes by extracts isolated from *Bacopa monniera* (L.) Wettst J Ethnopharmacol 2008 118 2 305 311 18534796 10.1016/j.jep.2008.04.017

[B36] Claria J Romano M Pharmacological intervention of cyclooxygenase-2 and 5-lipoxygenase pathways. Impact on inflammation and cancer Curr Pharm Des 2005 11 3431 3447 16250846 10.2174/138161205774370753

[B37] Gupta M Nath R Srivastava N Shanker K Kishor K Bhargava K Anti-inflammatory and antipyretic activities of β-sitosterol Planta Med 1980 39 2 157 163 6967611 10.1055/s-2008-1074919

[B38] Gupta R Sharma AK Dobhal MP Sharma MC Gupta RS Antidiabetic and antioxidant potential of β-sitosterol in streptozotocin-induced experimental hyperglycemia J Diabetes 2011 3 1 29 37 21143769 10.1111/j.1753-0407.2010.00107.x

[B39] Sroka Z Cisowski W Hydrogen peroxide scavenging, antioxidant and anti-radical activity of some phenolic acids Food Chem Toxicol 2003 41 6 753 758 12738180 10.1016/s0278-6915(02)00329-0

[B40] Vane JR Mechanism of action of nonsteroidal anti-inflammatory drugs Ameri J Med 1998 104 3 2S 8S 10.1016/s0002-9343(97)00203-9 9572314

[B41] Lapenna D Ciofani G Pierdomenico SD Neri M Cuccurullo C Giamberardino MA Cuccurullo F Inhibitory activity of salicylic acid on lipoxygenase-dependent lipid peroxidation Biochim Biophys Acta (BBA)-General Subjects 2009 1790 1 25 30 10.1016/j.bbagen.2008.09.007 18950686

[B42] Patel B Welch A Bingham S Luben R Day N Khaw K Lomas D Wareham N Dietary antioxidants and asthma in adults Thorax 2006 61 388 16467075 10.1136/thx.2004.024935 PMC2111195

[B43] Patrono C Rocca B Nonsteroidal antiinflammatory drugs: Past, present and future Pharmacol Res 2009 59 285 289 19416627 10.1016/j.phrs.2009.01.011

[B44] Chaudhary MA Imran I Bashir S Mehmood MH Rehman NU Gilani AH Evaluation of gut modulatory and bronchodilator activities of *Amaranthus spinosus* Linn BMC Complement Altern Med 2012 12 1 166 Epub ahead of print 23025418 10.1186/1472-6882-12-166 PMC3545920

[B45] Khan H Khan MA Gul F Hussain S Ashraf N **Anti-inflammatory activity of **** *Heliotropium strigosum * ****in animal models** Toxicol Ind Health 2013 **0748233713491813, first published on July 3, 2013** 10.1177/0748233713491813 23823617

[B46] Rehman NU Khan AU Alkharfy KM Gilani AH P**harmacological basis for the medicinal use of *****Lepidium sativum *****in airways disorders** Evid Based Complement Alternat Med 2012 10.1155/2012/596524 PMC3265128 22291849

